# A Comparative Analysis Applied to the Partial Discharges Identification in Dry-Type Transformers by Hall and Acoustic Emission Sensors

**DOI:** 10.3390/s22051716

**Published:** 2022-02-22

**Authors:** Bruno Albuquerque de Castro, Vitor Vecina dos Santos, Guilherme Beraldi Lucas, Jorge Alfredo Ardila-Rey, Rudolf Ribeiro Riehl, André Luiz Andreoli

**Affiliations:** 1 Department of Electrical Engineering, School of Engineering, São Paulo State University (UNESP), Bauru 17033-360, SP, Brazil; vitor.vecina@unesp.br (V.V.d.S.); guilherme.beraldi@unesp.br (G.B.L.); rudolf.riehl@unesp.br (R.R.R.); andre.andreoli@unesp.br (A.L.A.); 2 Department of Electrical Engineering, Universidad Técnica Federico Santa María, Av. Vicuña Mackenna 3939, Santiago de Chile 8940000, Chile; jorge.ardila@usm.cl

**Keywords:** dry-type insulated transformers, Hall-effect sensors, acoustic emission, pattern recognition, partial discharges

## Abstract

Dry-type insulated transformers stand out for their higher applicability in substations, high-voltage instrumentation systems, and electrical installations. In this machine, the insulation system is constituted of dielectric materials such as epoxy resin and Nomex paper. Some critical issues in the operation of this equipment, such as overload, moisture, or heat, can induce a slow degradation of the physical–chemical properties of the dielectric materials, which can culminate in the total failure of the transformer. However, before the transformer’s shutdown, it is common to detect discharge activity in the insulation system. Based on this issue, this work proposes an experimental and comparative analysis between acoustic emission and Hall-effect sensors, aiming at differentiating discharges in epoxy resin and Nomex paper, materials that constitute the insulation of the dry-type insulated transformers. Two signal processing techniques were studied: traditional frequency analysis and discrete wavelet transform. The objective is to develop signal processing techniques to differentiate each type of discharge since different discharges require different maintenance actions. The results obtained indicate that acoustic emission sensors and Hall sensors are promising in differentiating discharge in epoxy resin and Nomex paper. Furthermore, the pattern recognition tools presented by this work, which associated the wavelet levels energies and the energy of the full signals with the average band and the equivalent bandwidth, were effective to perform feature extraction of power transformer condition.

## 1. Introduction

The demand for power transformer monitoring systems has grown in recent years [1]. The goal is to reach a high level of control and prevention of total failures, improving the maintenance planning with punctual actions. Among the various existing topologies, dry-type transformers play a significant role in the electrical system as they are widely used in substations, instrumentation systems, electrical installations, and power system protection. The cooling is performed with natural air circulation, and the insulation is composed of solid dielectric materials such as paper and epoxy resin [2].

In practical scenarios, the transformers can suffer unexpected thermal, mechanical, electrical, and environmental stresses due to overload, moisture, heat, and other critical operational conditions. These issues induce a slow degradation of the machine’s insulation, leading the transformer to total failure [3,4,5,6]. In addition, failures in the impregnation of the epoxy resin can cause the formation of voids (air bubbles) and impair the capabilities of the insulation system [7,8].

In stages preceding total failure, it is usual to detect partial discharges (PDs) activity [9]. PDs are low-energy ionization processes of the dielectric materials that constitute the transformer insulation system (IEC 60270, 2000) [10], such as epoxy resin and Nomex paper [11]. In this sense, the identification of partial discharges is an important indication of the condition of the electrical machine. When discharges occur, they produce heat, light, electromagnetic, and acoustic waves, increasing the degradation of the dielectrics [9,12]. Therefore, the development of a partial discharge monitoring tool can prevent the transformers’ total failures. Among several methodologies [13,14,15,16,17], two promising techniques are related to current analysis and the acoustic emission technique. In the first, the behavior of the electric current is analyzed to identify the transients generated by PD activity [18,19,20]. In the second, piezoelectric transducers capture the acoustic waves emitted by the discharges [21,22,23]. More specifically, in the case of transformers with dry insulation, the failures can occur in the epoxy resin [24], which constitutes the device’s external insulation, and it is directly in contact with the environment. On the other hand, PDs can start in the paper that physically insulates the two transformer’s windings [25]. In this scenario, the development of methodologies that result in the differentiation of discharges in Nomex paper and epoxy resin becomes essential for improving the capabilities of the monitoring system since each type of material requires different maintenance actions.

This article aims to perform a comparative analysis between acoustic emission and Hall-effect sensors to perform the distinction of epoxy resin and Nomex paper partial discharge activity. Two signal processing techniques are presented. The first is based on traditional metrics such as energy, equivalent bandwidth, the average band of the current, and acoustic signals emitted by the two types of PDs. The second technique uses discrete wavelet transform to achieve the pattern recognition of the failures. Experimental results confirmed that all signal and sensing techniques presented a promising alternative to partial discharge recognition. However, the current analysis combined with the first set of metrics (energy, equivalent bandwidth, the average band) demonstrated a higher level of pattern recognition in relation to all studied techniques. Therefore, the contributions of this work are summarized as follows:

- Provide a comparative analysis between the sensitivity of Hall-effect and acoustic emission sensors to partial discharge detection in dry-type transformers.
- Improve the monitoring systems applied to dry-type transformers, by developing new signal processing approaches to perform pattern recognition between partial discharges in epoxy resin and Nomex paper.
- Make a comparative analysis of the two proposed algorithms to distinguish the discharges by using Hall-effect and acoustic emission sensors.

This article is divided into seven sections. Section 2 provides a discussion about partial discharges in dry-type transformers. The sensors based on Hall-effect and acoustic emission are discussed in Section 3. The signal processing approaches proposed by this work are presented in Section 4. The experimental setup and results are shown in Section 5 and Section 6, respectively. The conclusion of the article is presented in Section 7.

## 2. Partial Discharges in Dry-Type Transformers

Before a complete transformer insulation failure, it is common to detect low-energy ionization processes in an insulation system. This phenomenon, known as partial discharge (PD), can be considered an important indication of the operational condition of the dry transformer [25,26,27].

According to the IEC 60270 (2000) [10], partial discharge is defined as an electrical discharge that short-circuits specific regions of the dielectrics because of a local intensification of the electric field due to lower dielectric strength in the insulation system, which can be characterized by material degradation or voids. Among several developed techniques [9], the acoustic emission and current analysis have stood out as promising tools to perform partial discharge detection. In the first approach, piezoelectric transducers capture the acoustic emission of the PDs. In the second methodology, current sensors acquire the oscillations in the electrical current produced by these faults.

Although these approaches have successfully proved to be effective, the challenge is to develop signal processing tools that culminate with the pattern recognition of different types of failures by using acoustic and current analysis. For example, in dry-type transformers, there is a need to develop methodologies that can differentiate the partial discharge activity in Nomex and epoxy since the paper is used between the transformer windings and the resin insulates the rest of the device; hence, each fault requires different maintenance procedures.

In this context, several works have proposed methodologies to perform the partial discharge detection in epoxy resin and Nomex paper. Park et al. (2020) [24] studied the behavior of epoxy discharges in gas-insulated systems such as sulfur hexafluoride. Aakre et al. (2019) [7] developed a study related to the unconformities in the resin impregnation process, and proposed an equivalent circuit model to describe the effects of discharges on these materials. The three-capacitance and analytical models were proposed by Rodríguez-Serna et al. (2019) to evaluate the degradation of epoxy resins [28]. Nakamura et al. (2020) [26] assessed the electrical charge produced in epoxy materials when they are subjected to the effect of temperature.

In addition, several works have also evaluated the effect of discharges on insulating paper. Li et al. (2020) [25] presented a model to assess the degradation of insulating papers caused by partial discharges. Mishra et al. (2020) [29] proposed a method for evaluating the condition of insulating paper based on frequency domain spectroscopy (FDS) under temperature variations. The effects of moisture in Nomex-type papers was also the target of the studies proposed by Li et al. (2020) [30]. Although effective, the previously mentioned works did not perform the feature extraction aiming to differentiate discharges in resin and Nomex, materials that constitute the insulation of the dry transformer. In this sense, Ardila-Rey et al. (2018) proposed a data differentiation technique based on frequency analysis. The proposed algorithm, called the chromatic technique, proved to be effective in differentiating corona-type discharges and the discharges in bushings and Nomex paper [19]. Another work proposed by Ardila-Rey et al. (2021) differentiated surface discharges in Nomex paper and internal discharges in methacrylate-induced voids based on UHF tests [15].

As previously discussed, although current and acoustic emission techniques are widely used to perform partial discharge detection, there is a need to develop signal processing techniques that culminate in the differentiation of discharges in epoxy resin and Nomex paper. In this context, this article proposed two digital signal processing techniques based on Fourier and wavelet analysis which performed pattern recognition of these two failures. Furthermore, the sensitivity of piezoelectric and Hall-effect current sensors was assessed for partial discharge detection.

## 3. Piezoelectric and Hall-Effect Sensors

This section is divided into two subsections: the first presents the concepts of piezoelectricity, and the second the Hall-effect in current measurements.

### 3.1. Piezoelectric Sensors

Piezoelectric materials allow the conversion of mechanical energy into electrical energy (direct piezoelectric effect), and voltage into mechanical energy (reverse piezoelectric effect) [31]. Once partial discharges emit acoustic waves, which are characterized as mechanical waves, the piezoelectric transducers can be configured as sensors (direct piezoelectric effect) to acquire the frequencies emitted by a PD [32].

The basic constitutive relations of the direct and reverse piezoelectric effects for a piezoelectric material are given by Equations (1) and (2), respectively [33]:

(1) $$D_{i}=d_{ikl}T_{kl}+\varepsilon_{ik}^{T}E_{k}$$

(2) $$D_{ij}=S_{ijkl}^{E}T_{kl}+d_{kij}E_{k}$$

where $S_{ij}$ is the mechanical strain component; $d_{ikl}$ and $d_{kij}$ are the piezoelectric constants; $s_{ijkl}^{E}$ is the elastic compliance under a constant electric field; $T_{kl}$ is the mechanical stress component; $E_{k}$ and $D_{i}$ are the electric field and electrical displacement components, respectively; $\varepsilon_{ik}^{T}$ is the permittivity component at constant stress, and the subscripts ijkl represent the natural coordinate system of the piezoelectric crystal and take values of 1, 2, and 3.

Among several types of piezoelectric materials, the transducers based on PZT ceramics (Pb–lead zirconate titanate—lead zirconate titanate) have been gaining prominence due to their low cost and easy access.

### 3.2. Current Sensors

There are several types of current sensors, such as the shunt principle, high-frequency current transformers, Rogowski coils, Hall-effect sensors, and magnetoimpedance sensors.

For PD detection, the most common types are Rogowski coils, high-frequency current transformers, known as HFCTs, and inductive loop sensors (ILS), which are effective and have promising results in the field of transformers monitoring [34,35]. Basically, all of them consist of copper coils that surround a core of magnetic or nonmagnetic material. They capture current pulses from discharges by electromagnetic induction. This type of sensing has the advantage of not requiring galvanic contact. The sensor used in this work is made of a magnetic material core with coils coupled in series to a material that produces the Hall effect [36]. Although they have a lower frequency response [37], Hall-effect sensors are cheaper than high-frequency current transform (HFCT). The Hall effect can be explained by taking a metallic wire conducting an electric current under the action of a perpendicular magnetic field. The presence of the field induces deflection in the trajectory of the charges present in a semiconductor material. This change generates an electric field in the same direction as the new trajectory. Finally, a voltage, proportional to the electrical current, is generated due to the accumulation of charges at the ends of the Hall-effect material [36].

## 4. Signal Processing Analysis

As explained, the objective of this paper is to use the wavelet transform and frequency analysis techniques to characterize the two types of discharges by applying two types of sensors. Thus, this section is divided into two parts: frequency and wavelet analysis.

### 4.1. Frequency Analysis

The discrete Fourier transform (DFT) is a powerful tool used to obtain the frequency content of a signal. Taking a sequence with *N* samples, the DFT is defined as follows:

(3) $$X\left[k\right]=\sum_{n=0}^{N-1}x\left[n\right]e^{-j\frac{2\pi kn}{N}}$$

(4) $$\omega_{k}=\frac{2\pi k}{N},k=0,1,2,...,N-1$$

where *X* is the DFT of signal *x*, and $\omega_{k}$ is the frequency.

The chromatic technique (CT) was applied to characterize the frequency content of the two types of PD, considering the signals extracted from current and acoustic emission signals. CT is characterized as an effective pattern recognition tool, which allows feature extraction from complex signals [38,39]. The pattern recognition is performed by 3D maps that group similar features without the need for a supervised training process. This technique depends on the energy (E), the mean square frequency ($\omega_{c}$), and the equivalent bandwidth (B) and has never been applied in acoustic emission signals from partial discharge activity. Considering a given signal *x*[*n*] and its DFT magnitude and frequency, respectively $X_{n}$ and $\omega_{n}$, CT is defined as follows:

(5) $$E=\frac{1}{2\pi }\sum_{n=0}^{N-1}{\left|X_{n}\right|}^{2}$$

(6) $$\omega_{c}=\frac{\sum_{n=0}^{N-1}\omega_{n}{\left|X_{n}\right|}^{2}}{\sum_{n=0}^{N-1}{\left|X_{n}\right|}^{2}}$$

(7) $$B=\sqrt{\frac{1}{E}\sum_{n=0}^{N-1}{\left(\omega_{n}-\omega_{c}\right)}^{2}{\left|X_{n}\right|}^{2}}$$

where *E* is the energy of signal, $\omega_{c}$ is the mean square frequency, and *B* is the bandwidth.

The mean square frequency extract the center of the bandwidth of the spectrum. On the other hand, the equivalent bandwidth presents the real or effective bandwidth of the signals. Associated with the energy content, these parameters are a promising tool to perform data differentiation and were applied to distinguish partial discharges from epoxy and Nomex paper for each sensing technique applied.

### 4.2. Wavelet Analysis

The discrete wavelet transform (DWT) is a time–frequency domain tool that compresses and stretches a non-sinusoidal wave to decompose the original signal, creating a multiresolution of the original signal [40]. DWT scales are taken in the power of two, acting as a dual-band filter, which establishes the approximation and detail coefficients for several levels of decomposition. For the first level of a given sequence *x*[*n*], there is a high-pass filter and a low-pass filter, whose impulse responses are, respectively, $g_{1}\left[n\right]$ and $h_{1}\left[n\right]$, which produces the detail coefficients $d_{1}\left[k\right]$ and approximation coefficients $a_{1}\left[k\right]$, as shown in Equations (8) and (9) [41].

(8) $$d_{1}\left[k\right]=\sum_{n}g_{1}\left[n-2k\right]x\left[n\right]$$

(9) $$a_{1}\left[k\right]=\sum_{n}h_{1}\left[n-2k\right]x\left[n\right]$$

This operation can be performed repeatedly. After the first level, approximation and detail coefficients are extracted by decomposing the previous level, applying the impulse responses $g_{1}\left[n\right]$ and $h_{1}\left[n\right]$. Therefore, each level produces a sequence in which the sampling rate drops by half. Hence, the maximum number of wavelet levels is $\log_{2}\left(N\right)$ for a sequence with N samples. The approximation coefficients preserve the information related to low frequencies. On the other hand, detail coefficients retain high frequencies features. Some statistics can be applied to quantify wavelet levels. One of these metrics, used in this work, is the energy ($E_{w}$) of the coefficients (w[n]) of each wavelet level of detail or approximation, defined by

(10) $$E_{w}=\sum_{n=1}^{N}{\left|w[n]\right|}^{2}$$

The DWT was calculated for each type of discharge and sensor. The energies of wavelet levels were applied to form three-dimensional graphs in which each axis represents the energies of certain levels of approximation or detail. The objective was to present the WT as a data separation technique.

## 5. Methodology

This section is divided into two subsections. First, the experimental setup is presented, followed by the description of the signal processing methodologies of this study.

### 5.1. Experimental Setup

**Figure 1:**
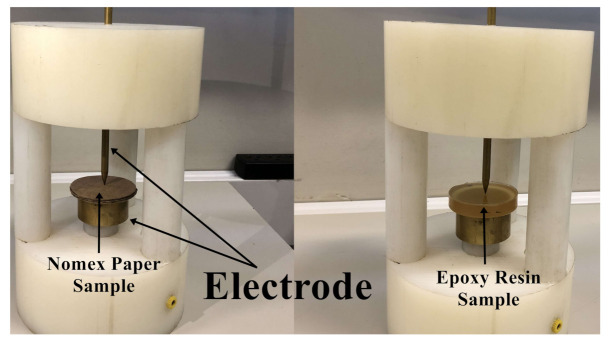
Electrode and material samples.

To investigate the effectiveness of the acoustic emission technique and Hall-effect current analysis on differentiating partial discharges in insulation materials of a dry transformer, several experiments were carried out on the samples of epoxy resin (Calas 92) and Nomex paper. The dimensions of the respective samples were 70 mm in diameter by 20 mm in thickness for the epoxy resin, and 70 mm in diameter by 6 mm in thickness for the insulating paper. A point-plan electrode was built to produce partial discharges in the samples of the materials, as shown in Figure 1. This electrode was supplied by a medium voltage source (General Electric 0–40 kVac) and inserted into a metallic box to emulate a protection wall, which is widely present in dry-type transformers.

A piezoelectric diaphragm, model 7BB-35-3, manufactured by Murata Electronics^®^ was coupled to the wall using liquid paraffin to capture the acoustic signals from PD activity. These transducers have a circular brass base 35 mm in diameter and 0.30 mm in thickness. The piezoelectric ceramic is the active element of 25 mm in diameter and 0.53 mm in thickness. This sensor has a frequency response up to 100 kHz according to [42]. The signal was amplified with a gain of 25 times, by the instrumentation amplifier model INA 128P (the Texas^®^), whose frequency response is up to 400 kHz.

A Tektronix^®^ Hall-effect based sensor, model A622, with a band in the range from 0 to 100 kHz and a maximum current of 100 A peak, was used to capture the current signals produced by the PDs. The sensitivity was set at 10 mV/A. The sensor wrapped the cable that fed the electrode to capture the failure activity.

**Figure 2:**
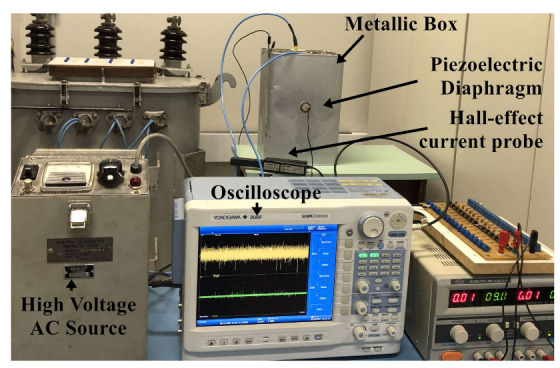
Experimental setup test bench.

The acquisition rate was set to 1 MHz to enhance Nyquist’s theorem. To avoid electromagnetic interference in the acoustic emission tests, the shield cables were grounded. In addition, the metallic box was grounded. Increasing voltage was applied to the material samples to elicit PD activity, which started at 10 kV for paper and 15 kV for epoxy resin. The electrode was positioned to touch the surface of the samples. No corona effect, breakdowns, or discharges were observed in other parts of the setup. The voltage level used in the tests promoted only surface discharges in the materials. Figure 2 presents the experimental setup test bench.

### 5.2. Signal Processing Analysis

**Figure 3:**
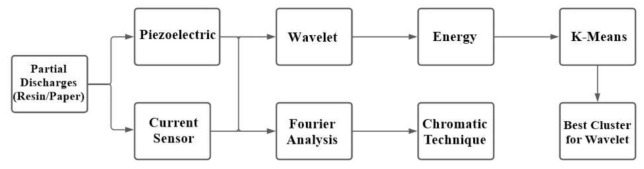
Flowchart of signal processing analysis.

For the frequency characterization of partial discharges, the average of 250 tests of fast Fourier transform of each component (resin and paper) was taken. All sequences were normalized to extract the average. After that, the CT parameters were calculated. The aim was to validate the effectiveness of the CT in the characterization of each type of PD considering current and acoustic emission signals. The CT parameters were constituted by a spatial coordinate value to form three-dimensional maps for data characterization. Wavelet analysis was performed by extracting energy from the levels of approximation and detail. Daubechies mother wavelet was chosen since it does not present discontinuities in its function. As the signals were acquired at one million samples per second, and considering a 0.1-s window on the oscilloscope, the number of samples of the current and acoustic signals were 100,000. Thus, the maximum number of wavelet levels was 16. The energy was calculated for each level of detail and approximation Therefore, each wavelet level (approximation and detail levels) presented 500 energy values (250 for epoxy resin tests and 250 for Nomex paper tests). To perform pattern recognition of discharges on paper and epoxy resin, three-dimensional maps were constituted with wavelet levels of approximation and detail. In this aspect, each axis of the Cartesian system corresponded to the 500 energy values of a single wavelet level. Taking each level of detail or approximation, combined three to three, there are 560 possibilities of separation maps. To simplify the presentation of the results, an algorithm was chosen to return the combination of levels with the lowest number of false diagnoses and whose maps were as far away as possible, considering the Euclidean distance of the mean coordinate of the “paper” and “epoxy” clusters. To simplify the presentation of results, only the best combinations of levels of approximation and detail were illustrated. Thus, two parameters were calculated: the average normalized Euclidean distance between the maps and the number of false diagnoses. This last parameter represents the number of discharge points occurring in epoxy resin that, erroneously, were presented as discharges in Nomex paper, and vice versa. The definition of these points of false diagnosis was given by the K-means algorithm [43]. The K-means algorithm was used to select the best result, which means the cluster of three wavelet levels that formed a 3D space of energy points, which returned the lowest errors and higher Euclidean distance between the data. These steps were performed for the two sensors. This way, it was possible to compare the effectiveness of each sensor in identifying the two types of discharges (epoxy or Nomex paper). A flowchart (Figure 3) summarizes the signal processing techniques used in this article.

## 6. Results and Discussion

This section is also divided into two subsections. First, the results related to piezoelectric transducer are presented, followed by the results and discussion produced by Hall-effect-based current sensor.

### 6.1. Acoustic Emission Analysis

**Figure 4:**
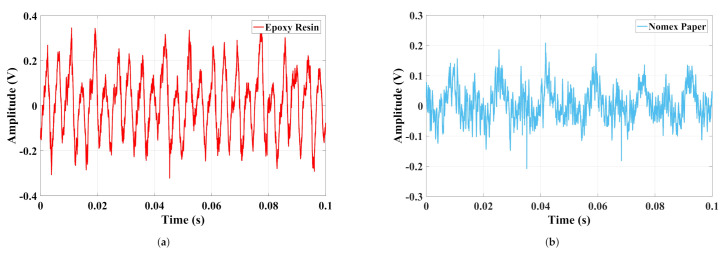
Time domain signals extracted from PDs in (**a**) epoxy resin and (**b**) Nomex paper.

In Figure 4, the acoustic signals in the time domain produced by discharges in epoxy resin and Nomex paper are presented.

**Figure 5:**
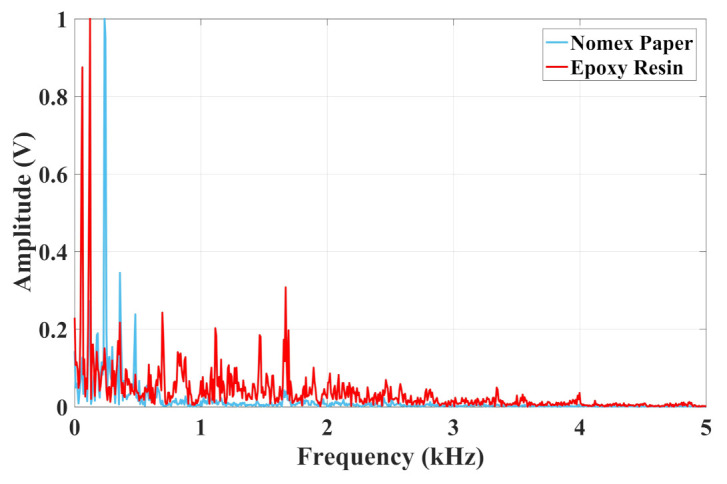
Frequency content of PD activity in epoxy resin and Nomex paper for piezoelectric transducer.

It can be observed that the acoustic emission signals from discharges in Nomex paper have small amplitudes concerning epoxy resin. This difference was 0.15 V, considering that the maximum value for resin was 0.35 V and 0.2 V for Nomex paper. To verify the frequency content of the signals, Figure 5 presents the normalized spectrum of the discharges in Nomex paper and epoxy resin.

As seen in Figure 5, the frequency content for epoxy and Nomex discharges occurred at frequencies up to 5 kHz. For the epoxy material, the maximum value was at 120 Hz and the frequency band was concentrated between 500 Hz and 2 kHz. Concerning the Nomex paper, the peak value was at 240 Hz, and the frequency content was concentrated until 1 kHz.

**Figure 6:**
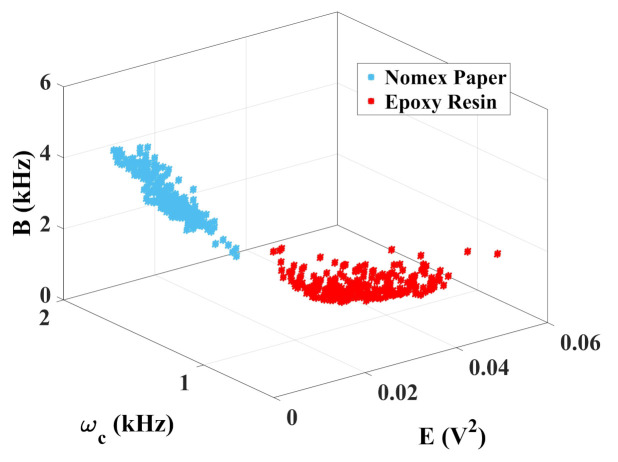
Chromatic technique for piezoelectric transducer.

To evaluate the effectiveness of the chromatic technique for discharge differentiation, the 3D color maps were generated as shown in Figure 6.

As noted, the chromatic technique performed the correct diagnosis, since two well-defined regions can be verified for each case of PD. For epoxy discharges, the average band remained between 530 Hz and 680 Hz. For Nomex, the values were between 1 kHz and 1.5 kHz. The energy of the signals for discharges on Nomex paper remained between 0.01 V${}^{2}$ and 0.02 V${}^{2}$, while for epoxy resin these values were between 0.02 V${}^{2}$ and 0.04 V${}^{2}$. It is also observed that the equivalent bandwidth varied from 1 kHz to 4 kHz for Nomex paper and from 1 kHz to 2 kHz for the resin. The normalized Euclidean distance between the center of the two regions was 0.33 units. For these calculations, the values of the two sets were normalized and the distance between the centroids was calculated.

**Figure 7:**
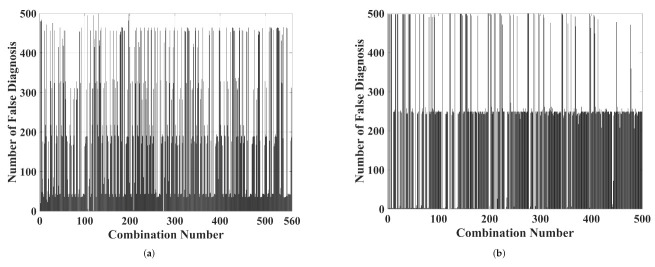
The number of false diagnoses for each combination (presented in the Appendix A) of three levels for (**a**) approximation and (**b**) detail coefficients.

Regarding the wavelet analysis, three-dimensional graphics such as those presented by the chromatic technique were carried out. Each axis was formed by the energy of levels of approximation and detail. As previously stated, the number of possible combinations for each level was 560 (presented in the Appendix A). To simplify the results’ presentation, Figure 7 shows the number of false diagnoses generated for each combination. False diagnoses were defined as overlaps between color maps formed by discharges on epoxy resin and those generated by discharges in Nomex paper.

**Figure 8:**
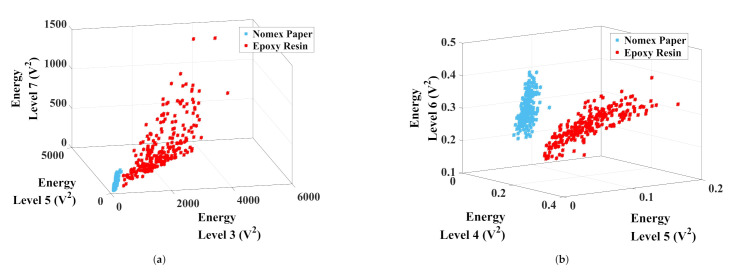
Clusters formed by the best combinations of (**a**) approximation and (**b**) detail coefficients.

The best combination for data separation appointed by the K-means algorithm was the energy of the approximation levels 3, 5, and 7. For the detail analysis, the best combination was for levels 4, 5, and 6. Regarding the approximation analysis (Figure 8a), level 3 remained between 100 V${}^{2}$ and 400 V${}^{2}$ and between 500 V${}^{2}$ and 3800 V${}^{2}$ for Nomex and epoxy discharges, respectively. The wavelet 5 level varied from 90 V${}^{2}$ to 320 V${}^{2}$ for Nomex paper discharges and from 600 V${}^{2}$ to 3700 V${}^{2}$ for epoxy resin discharges. For level 7, these values remained between 40 V${}^{2}$ and 253 V${}^{2}$ for Nomex and between 44 V${}^{2}$ and 790 V${}^{2}$ for epoxy. The normalized distance between the clusters was 0.39, forming two well-defined regions that did not present false diagnoses. The same analysis was performed for the levels of detail (Figure 8b). The level of detail 4 remained between 0.07 V${}^{2}$ and 1 V${}^{2}$ and between 0.13 V${}^{2}$ and 0.28 V${}^{2}$ for Nomex paper and epoxy discharges, respectively. The wavelet 5 level varied between 0.04 V${}^{2}$ and 0.07 V${}^{2}$ for the discharges on Nomex and between 0.06 V${}^{2}$ and 0.17 V${}^{2}$ for the resin discharges. For level 6, these values remained between 0.2 V${}^{2}$ and 0.4 V${}^{2}$ for the paper and between 0.16 V${}^{2}$ and 0.30 V${}^{2}$ for the resin. The normalized distance between the clusters was 0.36, forming two well-defined regions that did not present false diagnoses as well.

### 6.2. Current Analysis

**Figure 9:**
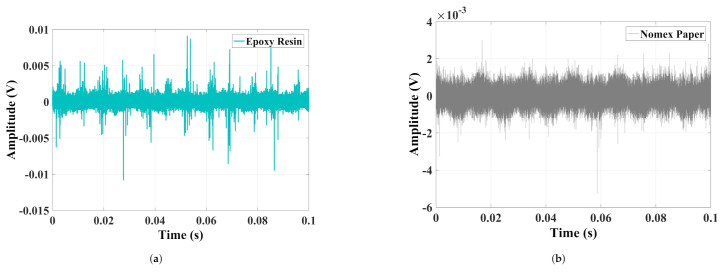
Time domain signals extracted from PDs in (**a**) epoxy resin and (**b**) Nomex paper.

In Figure 9, the current signals in the time domain produced by discharges in epoxy resin and Nomex paper are presented.

Figure 9, verifies that, as for the acoustic emission signals, the discharges in Nomex paper produced lower current amplitudes than the values presented by the discharges in epoxy resin.

**Figure 10:**
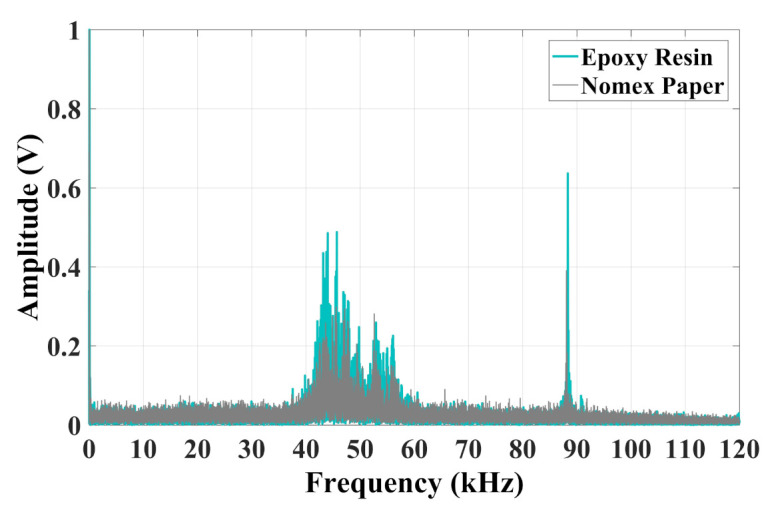
Frequency content of PD activity in epoxy resin and Nomex paper for Hall-effect current sensor.

In Figure 10 the frequency spectrum of current signals is presented. For both types of discharges, the maximum value was presented at the frequency of 60 Hz. However, it was also observed that, for both cases, the frequency band had a predominance between 40 kHz and 60 kHz, with a peak around 90 kHz. The discharges in epoxy resin presented higher peaks for all frequencies.

**Figure 11:**
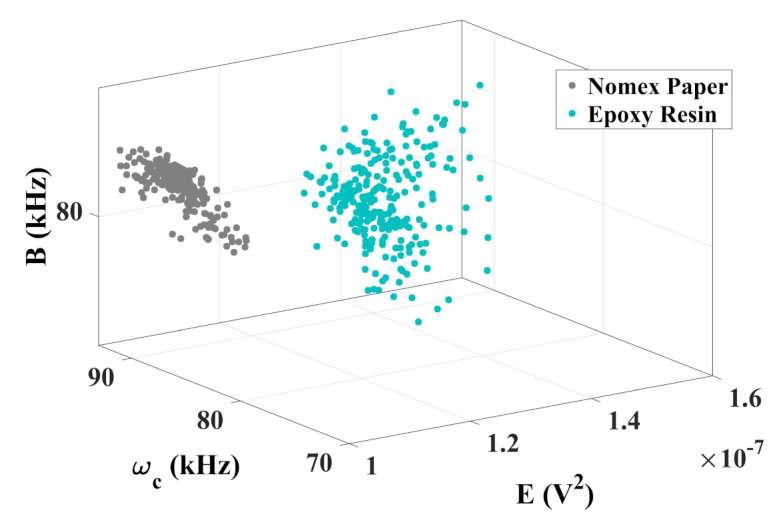
Chromatic technique for Hall-effect current sensor.

The chromatic technique is presented in Figure 11.

According to Figure 11, the chromatic technique performed the correct diagnosis of each type of discharge, since the color maps formed two well-distinct regions representing the partial discharges that occurred on Nomex paper and epoxy resin. Thus, the current sensor used in this work can be a promising alternative to differentiate types of discharges.

In the case of epoxy PDs, the average band values remained between 78 kHz and 88 kHz. For Nomex, these values were between 88 kHz and 92 kHz. It can be seen that the energy of the signals for Nomex discharges remained between $1\times 10^{-7}$ V${}^{2}$ and $1.13\times 10^{-7}$ V${}^{2}$, while for epoxy resin, these values were between $1.2\times 10^{-7}$ V${}^{2}$ and $1.5\times 10^{-7}$ V${}^{2}$. It is also observed that Equivalent bandwidth varied from 78 kHz to 82 kHz for Nomex and from 79 kHz to 83 kHz for epoxy. The normalized Euclidean distance between the center of the two regions was 0.41 units.

**Figure 12:**
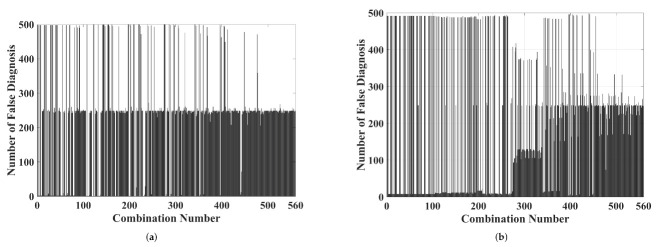
The number of false diagnoses for each combination (presented in the Appendix A) of three levels for (**a**) approximation and (**b**) detail coefficients.

The same wavelet analysis performed for piezoelectric transducer was achieved. Figure 12 presents the number of false diagnoses for each combination of three wavelet levels.

**Figure 13:**
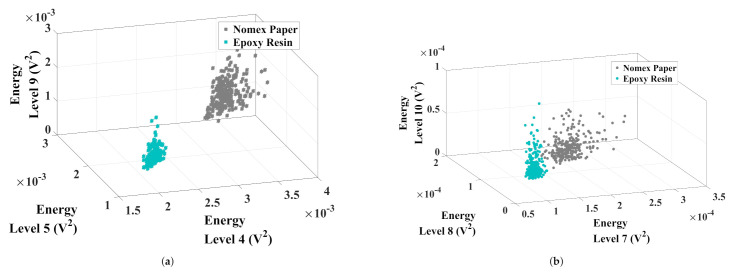
Clusters formed by the best combinations of (**a**) approximation and (**b**) detail coefficients.

The best combination for data separation appointed by the K-means algorithm was the energy of the approximation levels 4, 5, and 9. For the detail analysis, the best combination was for levels 7, 8, and 10. In Figure 13, the maps formed by the best results for approximation and detail coefficients are presented.

Concerning the approximation analysis (Figure 13a), level 4 remained around 2 mV${}^{2}$ for epoxy discharges and between 2.5 mV${}^{2}$ and 3.8 mV${}^{2}$ for Nomex discharges. The wavelet 5 level was around 1.5 mV${}^{2}$ for epoxy resin and between 2.5 mV${}^{2}$ and 3.6 mV${}^{2}$ for Nomex paper discharges. For level 9, these values remained between 0.6 mV${}^{2}$ and 1 mV${}^{2}$ for resin and from 1 mV${}^{2}$ to 2 mV${}^{2}$ for Nomex paper. The normalized distance between clusters was 0.40, forming two well-defined regions.

Regarding the detail analysis (Figure 13b), level 7 remained at 0.1 mV${}^{2}$ and between 0.1 mV${}^{2}$ and 0.2 mV${}^{2}$ for discharges in epoxy resin and Nomex paper, respectively. The wavelet 8 level ranged between 0.07 mV${}^{2}$ and 1 mV${}^{2}$ for discharges in Nomex and from 0.12 mV${}^{2}$ to 0.18 mV${}^{2}$ for epoxy resin discharges. For level 10, these values remained between 0.02 mV${}^{2}$ and 0.06 mV${}^{2}$ for Nomex paper, and between 0.01 mV${}^{2}$ and 0.08 mV${}^{2}$ for epoxy material. For the detail coefficient analysis, the normalized distance between the clusters was 0.36.

### 6.3. Comparative Analysis

In order to carry out a comparative analysis between the sensors and the proposed signal processing techniques, the normalized Euclidean distances are presented in Table 1.

Regarding the application of the piezoelectric sensor, the Euclidian distances produced by the application of the chromatic technique, approximation, and detail analysis were, respectively, 0.39, 0.39, and 0.36. On the other hand, for the current sensor, the same distances were, respectively, 0.41, 0.40, and 0.36. By analyzing Table 1, it is verified that chromatic technique combined with the current sensor demonstrated the best alternative to performing data separation from partial discharge activity since it presented the highest value for Euclidian distance (0.41). The smallest Euclidian distance was found for the detail coefficients, which was 0.36 units for piezoelectric and Hall sensors.

Although the chromatic technique combined with the Hall-effect probe presented higher Euclidian distances, all color maps formed by frequency and discrete wavelet transform demonstrated great potential for the diagnosis of the type of partial discharge in a dry-type transformer since they presented well-defined regions for each type of discharge.

## 7. Conclusions

Among several topologies, dry-type transformers play an important role in the electrical system, and their monitoring is crucial. Failures can occur in the epoxy resin, which constitutes the transformer’s external insulation and is directly in contact with the environment. On the other hand, PDs can start in the papers that insulate the two transformer’s windings. In this context, the differentiation between discharges in Nomex paper and epoxy resin becomes essential for improving the monitoring system capabilities, since each type of material requires different maintenance actions or planning. Therefore, based on acoustic emission and electrical current tests, this work presented two techniques of digital signal processing to establish differences between the partial discharges occurring in two types of materials commonly found in the insulation system of the dry-type transformer. A point-plan electrode was carried out to induce surface discharges in the samples, but other topologies can be further analyzed.

From the results obtained, it is verified that the chromatic technique and the energy of the wavelet levels were effective in differentiating the discharges. Although the low-cost sensors used in this work provided promising results, their frequency content is lower compared to traditional sensors. However, this work demonstrated that the piezoelectric sensor and the Hall-effect probe could be alternatives to monitoring systems applied to dry-type transformers. Furthermore, new technologies related to microelectronics are increasing the frequency response of Hall-effect materials and can be applied in future works [44].

## Figures and Tables

**Table 1:** Normalized Euclidean distances for each technique.

Technique	Sensor	
	Piezoelectric	Hall-Effect
Chromatic	0.39	0.41
Energy of approximation coefficients	0.39	0.40
Energy of detail coefficients	0.36	0.36

## Data Availability

Not applicable.

## Appendix A

**Table A1:** Wavelet Levels combinations.

C	WL			C	WL			C	WL			C	WL			C	WL			C	WL		
1	1	2	3	101	1	13	15	201	3	4	9	301	4	7	13	400	6	7	13	501	8	13	16
2	1	2	4	102	1	13	16	202	3	4	10	302	4	7	14	401	6	7	14	502	8	14	15
3	1	2	5	103	1	14	15	203	3	4	11	303	4	7	15	402	6	7	15	503	8	14	16
4	1	2	6	104	1	14	16	204	3	4	12	304	4	7	16	403	6	7	16	504	8	15	16
5	1	2	7	105	1	15	16	205	3	4	13	305	4	8	9	404	6	8	9	505	9	10	11
6	1	2	8	106	2	3	4	206	3	4	14	306	4	8	10	405	6	8	10	506	9	10	12
7	1	2	9	107	2	3	5	207	3	4	15	307	4	8	11	406	6	8	11	507	9	10	13
8	1	2	10	108	2	3	6	208	3	4	16	308	4	8	12	407	6	8	12	508	9	10	14
9	1	2	11	109	2	3	7	209	3	5	6	309	4	8	13	408	6	8	13	509	9	10	15
10	1	2	12	110	2	3	8	210	3	5	7	310	4	8	14	409	6	8	14	510	9	10	16
11	1	2	13	111	2	3	9	211	3	5	8	311	4	8	15	410	6	8	15	511	9	11	12
12	1	2	14	112	2	3	10	212	3	5	9	312	4	8	16	411	6	8	16	512	9	11	13
13	1	2	15	113	2	3	11	213	3	5	10	313	4	9	10	412	6	9	10	513	9	11	14
14	1	2	16	114	2	3	12	214	3	5	11	314	4	9	11	413	6	9	11	514	9	11	15
15	1	3	4	115	2	3	13	215	3	5	12	315	4	9	12	414	6	9	12	515	9	11	16
16	1	3	5	116	2	3	14	216	3	5	13	316	4	9	13	415	6	9	13	516	9	12	13
17	1	3	6	117	2	3	15	217	3	5	14	317	4	9	14	416	6	9	14	517	9	12	14
18	1	3	7	118	2	3	16	218	3	5	15	318	4	9	15	417	6	9	15	518	9	12	15
19	1	3	8	119	2	4	5	219	3	5	16	319	4	9	16	418	6	9	16	519	9	12	16
20	1	3	9	120	2	4	6	220	3	6	7	320	4	10	11	419	6	10	11	520	9	13	14
21	1	3	10	121	2	4	7	221	3	6	8	321	4	10	12	420	6	10	12	521	9	13	15
22	1	3	11	122	2	4	8	222	3	6	9	322	4	10	13	421	6	10	13	522	9	13	16
23	1	3	12	123	2	4	9	223	3	6	10	323	4	10	14	422	6	10	14	523	9	14	15
24	1	3	13	124	2	4	10	224	3	6	11	324	4	10	15	423	6	10	15	524	9	14	16
25	1	3	14	125	2	4	11	225	3	6	12	325	4	10	16	424	6	10	16	525	9	15	16
26	1	3	15	126	2	4	12	226	3	6	13	326	4	11	12	425	6	11	12	526	10	11	12
27	1	3	16	127	2	4	13	227	3	6	14	327	4	11	13	426	6	11	13	527	10	11	13
28	1	4	5	128	2	4	14	228	3	6	15	328	4	11	14	427	6	11	14	528	10	11	14
29	1	4	6	129	2	4	15	229	3	6	16	329	4	11	15	428	6	11	15	529	10	11	15
30	1	4	7	130	2	4	16	230	3	7	8	330	4	11	16	429	6	11	16	530	10	11	16
31	1	4	8	131	2	5	6	231	3	7	9	331	4	12	13	430	6	12	13	531	10	12	13
32	1	4	9	132	2	5	7	232	3	7	10	332	4	12	14	431	6	12	14	532	10	12	14
33	1	4	10	133	2	5	8	233	3	7	11	333	4	12	15	432	6	12	15	533	10	12	15
34	1	4	11	134	2	5	9	234	3	7	12	334	4	12	16	433	6	12	16	534	10	12	16
35	1	4	12	135	2	5	10	235	3	7	13	335	4	13	14	434	6	13	14	535	10	13	14
36	1	4	13	136	2	5	11	236	3	7	14	336	4	13	15	435	6	13	15	536	10	13	15
37	1	4	14	137	2	5	12	237	3	7	15	337	4	13	16	436	6	13	16	537	10	13	16
38	1	4	15	138	2	5	13	238	3	7	16	338	4	14	15	437	6	14	15	538	10	14	15
39	1	4	16	139	2	5	14	239	3	8	9	339	4	14	16	438	6	14	16	539	10	14	16
40	1	5	6	140	2	5	15	240	3	8	10	340	4	15	16	439	6	15	16	540	10	15	16
41	1	5	7	141	2	5	16	241	3	8	11	341	5	6	7	440	7	8	9	541	11	12	13
42	1	5	8	142	2	6	7	242	3	8	12	342	5	6	8	441	7	8	10	542	11	12	14
43	1	5	9	143	2	6	8	243	3	8	13	343	5	6	9	442	7	8	11	543	11	12	15
44	1	5	10	144	2	6	9	244	3	8	14	344	5	6	10	443	7	8	12	544	11	12	16
45	1	5	11	145	2	6	10	245	3	8	15	345	5	6	11	444	7	8	13	545	11	13	14
46	1	5	12	146	2	6	11	246	3	8	16	346	5	6	12	445	7	8	14	546	11	13	15
47	1	5	13	147	2	6	12	247	3	9	10	347	5	6	13	446	7	8	15	547	11	13	16
48	1	5	14	148	2	6	13	248	3	9	11	348	5	6	14	447	7	8	16	548	11	14	15
49	1	5	15	149	2	6	14	249	3	9	12	349	5	6	15	448	7	9	10	549	11	14	16
50	1	5	16	150	2	6	15	250	3	9	13	350	5	6	16	449	7	9	11	550	11	15	16
51	1	6	7	151	2	6	16	251	3	9	14	351	5	7	8	450	7	9	12	551	12	13	14
52	1	6	8	152	2	7	8	252	3	9	15	352	5	7	9	451	7	9	13	552	12	13	15
53	1	6	9	153	2	7	9	253	3	9	16	353	5	7	10	452	7	9	14	553	12	13	16
54	1	6	10	154	2	7	10	254	3	10	11	354	5	7	11	453	7	9	15	554	12	14	15
55	1	6	11	155	2	7	11	255	3	10	12	355	5	7	12	454	7	9	16	555	12	14	16
56	1	6	12	156	2	7	12	256	3	10	13	356	5	7	13	455	7	10	11	556	12	15	16
57	1	6	13	157	2	7	13	257	3	10	14	357	5	7	14	456	7	10	12	557	13	14	15
58	1	6	14	158	2	7	14	258	3	10	15	358	5	7	15	457	7	10	13	558	13	14	16
59	1	6	15	159	2	7	15	259	3	10	16	359	5	7	16	458	7	10	14	559	13	15	16
60	1	6	16	160	2	7	16	260	3	11	12	360	5	8	9	459	7	10	15	560	14	15	16
61	1	7	8	161	2	8	9	261	3	11	13	361	5	8	10	460	7	10	16				
62	1	7	9	162	2	8	10	262	3	11	14	362	5	8	11	461	7	11	12				
63	1	7	10	163	2	8	11	263	3	11	15	363	5	8	12	462	7	11	13				
64	1	7	11	164	2	8	12	264	3	11	16	364	5	8	13	463	7	11	14				
65	1	7	12	165	2	8	13	265	3	12	13	365	5	8	14	464	7	11	15				
66	1	7	13	166	2	8	14	266	3	12	14	366	5	8	15	465	7	11	16				
67	1	7	14	167	2	8	15	267	3	12	15	367	5	8	16	466	7	12	13				
68	1	7	15	168	2	8	16	268	3	12	16	368	5	9	10	467	7	12	14				
69	1	7	16	169	2	9	10	269	3	13	14	369	5	9	11	468	7	12	15				
70	1	8	9	170	2	9	11	270	3	13	15	370	5	9	12	469	7	12	16				
71	1	8	10	171	2	9	12	271	3	13	16	371	5	9	13	470	7	13	14				
72	1	8	11	172	2	9	13	272	3	14	15	372	5	9	14	471	7	13	15				
73	1	8	12	173	2	9	14	273	3	14	16	373	5	9	15	472	7	13	16				
74	1	8	13	174	2	9	15	274	3	15	16	374	5	9	16	473	7	14	15				
75	1	8	14	175	2	9	16	275	4	5	6	375	5	10	11	474	7	14	16				
76	1	8	15	176	2	10	11	276	4	5	7	376	5	10	12	475	7	15	16				
77	1	8	16	177	2	10	12	277	4	5	8	377	5	10	13	476	8	9	10				
78	1	9	10	178	2	10	13	278	4	5	9	378	5	10	14	477	8	9	11				
79	1	9	11	179	2	10	14	279	4	5	10	379	5	10	15	478	8	9	12				
80	1	9	12	180	2	10	15	280	4	5	11	380	5	10	16	479	8	9	13				
81	1	9	13	181	2	10	16	281	4	5	12	381	5	11	12	480	8	9	14				
82	1	9	14	182	2	11	12	282	4	5	13	382	5	11	13	481	8	9	15				
83	1	9	15	183	2	11	13	283	4	5	14	383	5	11	14	482	8	9	16				
84	1	9	16	184	2	11	14	284	4	5	15	384	5	11	15	483	8	10	11				
85	1	10	11	185	2	11	15	285	4	5	16	385	5	11	16	484	8	10	12				
86	1	10	12	186	2	11	16	286	4	6	7	386	5	12	13	485	8	10	13				
87	1	10	13	187	2	12	13	287	4	6	8	387	5	12	14	486	8	10	14				
88	1	10	14	188	2	12	14	288	4	6	9	388	5	12	15	487	8	10	15				
89	1	10	15	189	2	12	15	289	4	6	10	389	5	12	16	488	8	10	16				
90	1	10	16	190	2	12	16	290	4	6	11	390	5	13	14	489	8	11	12				
91	1	11	12	191	2	13	14	291	4	6	12	391	5	13	15	490	8	11	13				
92	1	11	13	192	2	13	15	292	4	6	13	392	5	13	16	491	8	11	14				
93	1	11	14	193	2	13	16	293	4	6	14	393	5	14	15	492	8	11	15				
94	1	11	15	194	2	14	15	294	4	6	15	394	5	14	16	493	8	11	16				
95	1	11	16	195	2	14	16	295	4	6	16	395	5	15	16	494	8	12	13				
96	1	12	13	196	2	15	16	296	4	7	8	396	6	7	8	495	8	12	14				
97	1	12	14	197	3	4	5	297	4	7	9	397	6	7	9	496	8	12	15				
98	1	12	15	198	3	4	6	298	4	7	10	398	6	7	10	497	8	12	16				
99	1	12	16	199	3	4	7	299	4	7	11	399	6	7	11	498	8	13	14				
100	1	13	14	200	3	4	8	300	4	7	12	400	6	7	12	499	8	13	15				

WL —Wavelt Levels; C—Combinations.
